# Understanding university students' attitudes and preferences for internet-based mental health interventions

**DOI:** 10.1016/j.invent.2024.100722

**Published:** 2024-02-06

**Authors:** Ömer Özer, Burak Köksal, Ahmet Altinok

**Affiliations:** a Department of Social Work and Consultancy, Open Education Faculty, Anadolu University, Eskisehir, Turkiye; b Counseling and Guidance Center, Gaziosmanpaşa University, Tokat, Turkiye; c Department of Psychology, Experimental Psychology, University of Groningen, Groningen, the Netherlands

**Keywords:** Internet-based interventions, Digital literacy, Attitudes towards internet-based interventions, Psychotherapy preferences, E-health

## Abstract

Internet-based interventions are recognised as a practical approach to address mental health issues. The acceptance and utilisation of such interventions are closely linked to user attitudes and preferences. This study aims to examine the predictors of university students' attitudes towards internet-based interventions. Additionally, it seeks to elucidate students' preferences regarding crucial features of these interventions, such as the format, delivery mode, content type, and structural components, to understand better what makes these interventions appealing and practical for university students.

A total of 273 university students (comprising 68 % females and 32 % males) participated in the study. The data collection instruments employed were the Personal Information Form, Internet-Based Intervention Preference Survey, E-therapy Attitude Measure (ETAM), Digital Literacy Scale, Patient Health Questionnaire-9, and the Generalized Anxiety Disorder-7 (GAD-7). The data were analysed utilising descriptive statistics, Pearson correlation analysis, and multiple linear regression analysis.

The multiple regression analysis revealed digital literacy as a predictive factor for attitudes towards internet-based interventions. Demographic variables, such as age and gender, and psychological variables, such as depression and anxiety levels, were found not to be associated with attitudes towards these interventions.

While students are actively seeking mental health information online, a significant majority remain unaware of internet-based interventions. They show a preference for interventions offering greater human interaction, including face-to-face guidance and video content featuring people. Participants favour completing one or two sessions of the intervention weekly. Desired features of internet-based interventions include self-assessment scales, relatable characters, voice relaxation exercises, practical daily life activity tasks, and weekly reminders throughout the process.

In conclusion, initiatives aimed at enhancing digital literacy levels could foster more positive attitudes towards internet-based interventions among students. Developers creating Internet-Based Interventions (IBI) for university students should consider these preferences.

## Highlights

• Despite active online searches for mental health information, a significant portion of students remain unaware of internet-based interventions.
• Students show a strong preference for internet-based interventions that offer higher levels of human interaction, including face-to-face guidance and human-centered video content.
• Enhancing digital literacy can foster more positive attitudes towards internet-based interventions among university students.

## 1 Introduction

Mental health problems, particularly depression and anxiety, are quite prevalent among university students (Akhtar et al., 2020; Gao et al., 2020; Garlow et al., 2008; Hunt and Eisenberg, 2010; Jenkins et al., 2021). Almost half of the students are reported to face issues at a diagnosable level within a one-year period (Blanco et al., 2008). Considering that issues like depression, anxiety, and stress typically commence during adolescence and peak at 25 years of age, it's evident that university students are at risk regarding their mental health (Kessler et al., 2007). According to the studies conducted by the World Health Organisation in different countries with wide participation and evaluating the prevalence of mental disorders in university students, it has been observed that mental health problems have become widespread; 35 % of the participants met the criteria for at least one of the common disorders whose lifetime prevalence was evaluated, and 31 % of the participants met the criteria for at least one of the disorders when the 12-month prevalence was evaluated (Auerbach et al., 2016, Auerbach et al., 2018).

Despite the prevalence of mental health problems, university students' propensity to seek help is not consistent. Many students are often reluctant to seek help, and their help-seeking behaviours are limited due to obstacles such as stigma, lack of qualified professionals, and economic reasons (Bruffaerts et al., 2019; Czyz et al., 2013; Ebert et al., 2019; Eisenberg et al., 2009; Marsh and Wilcoxon, 2015; Punton et al., 2022).

Internet-based interventions, considering their advantages such as reaching more individuals, reducing waiting list loads, allowing individuals to work at their own pace (Cuijpers and Schuurmans, 2007), easy access, time and cost savings (Linardon et al., 2021), possibility to revisit, monitoring clients' progress using software features (Andersson and Titov, 2014), reducing stigma by maintaining anonymity (Gericke et al., 2021), and fostering a more comfortable environment to express themselves openly and honestly (Chan et al., 2016a), are suitable tools for offering psychological services to university students. The effectiveness of internet-based interventions among university students has been studied in various research concerning stress (Amanvermez et al., 2021; Harrer et al., 2018; Sun et al., 2022), alcohol and substance use (Hustad et al., 2010; Kypri et al., 2009), social anxiety (Kählke et al., 2019; McCall et al., 2018), anxiety and depression (McCloud et al., 2020; Newman et al., 2021), and eating disorders (Bauer et al., 2009; Harrer et al., 2019). A meta-analysis that included eighty-nine studies has indicated that internet-based interventions could be effective in improving depression, anxiety, and mental health among university students. However, there is a need for more research to uncover the mechanisms of these interventions (Lattie et al., 2019).

Users' acceptance and practical usage of these widely studied internet-based interventions are associated with their attitudes towards such interventions (Apolinário-Hagen et al., 2022; Linardon et al., 2020, Linardon et al., 2021). However, studies evaluating university students' attitudes and preferences towards internet-based interventions are limited. A study assessing university students' intentions and use of internet-based self-help resources (Levin et al., 2016) indicated that students have low interest in using these resources and face barriers like stigma, privacy, and confidentiality concerns. Another study (Chan et al., 2016a) highlighted similar concerns but also pointed out reduced stigma and ease of access as advantages.

Digital/computer literacy is another variable affecting the acceptance and use of internet-based mental health interventions. Digital literacy is characterised by having the necessary digital skills to use and guide internet-focused technology and feeling comfortable or eager to use digital tools. Lower digital literacy is seen as a barrier to internet-based interventions (Chiu et al., 2009). These competencies are also essential predictors for using eHealth applications (Kontos et al., 2014).

An essential variable affecting psychological aid processes is the preferences of the counselees. Taking into account the preferences of the counselees in psychological aid services is considered a golden standard (APA Presidential Task Force on Evidence-Based Practice, 2006). Experts should provide services sensitive to the preferences of the counselees. In this context, considering preferences in the psychological aid process is the initial step to offering personalised aid. Individuals receiving help according to their psychological counseling preferences tend to have higher engagement in the process, more favourable outcomes, and lower early termination rates (Goates-Jones and Hill, 2008; Swift et al., 2018; Swift and Callahan, 2009). In their study evaluating adults' preferences towards internet-based interventions, Batterham and Calear (2017) reported that participants preferred accessing through computers instead of mobile devices and face-to-face processes over online resources. It stated that women, younger individuals, and people with higher education levels were more likely to prefer internet-based interventions. In a study assessing preferences for depression treatment, Dorow et al. (2018) highlighted that one in five participants preferred internet-based interventions, and this group was mainly comprised of younger individuals with higher education levels. During the development process of an alcohol reduction intervention aimed at young adults, it is stated that users preferred an application that would work more on mobile devices, preferred to record alcohol consumption, mood statuses, and treatment objectives, and favored animation videos and pastel colors (Schouten et al., 2023). Previous studies have primarily assessed the preferences of adults; however, the preferences of university students, arguably the group with the highest likelihood of using internet-based interventions, have not been clearly established in the literature.

Proceeding from here, we believe revealing university students' attitudes and preferences towards internet-based interventions will enhance the acceptability and effectiveness of the platforms to be developed. In this study, we aimed to uncover the attitudes and preferences of university students towards internet-based interventions. We believe the findings will contribute to planning the content and presentation of intervention programs to be developed specifically for university students. Moreover, internet-based interventions are predominantly studied in developed Western countries. Internet-based interventions are relatively new in Turkey, and there are a limited number of studies on this topic (Akin-Sari et al., 2022a; Akin-Sari et al., 2022b; Göcek-Yorulmaz, 2020; Özer et al., 2023). We envision this study serving as a starting point for internet-based interventions to be developed in Turkey and similar countries in the forthcoming periods.

## 2 Method

### 2.1 Data collection

The university website announced the study, and posters hung in all faculties. Participants were informed that they could optionally provide their email addresses, and at the end of the data collection process, a lottery would be conducted using these email addresses to give three participants gift vouchers worth 100 TL to be used for buying books. Sharing the email address was not made mandatory. The data was collected using the Google Forms service. The data collection link was kept open from March 15, 2023, to May 15, 2023. Before the data collection process, ethical committee approval for the research was obtained from the Tokat Gaziosmanpaşa University Social and Humanities Research Ethical Committee (Date: March 7, 2023/ Decision No: 04.06).

### 2.2 Participants

A total of 273 participants were included in the study. The participants' ages ranged from 18 to 47 years, with a mean age of 22.41 (SD = 5.47). Of the participants, 68 % were female (*n* = 187) and 32 % were male (*n* = 86). The sample was distributed across grades, with 40 % in grade 1, 29 % in grade 2, 15 % in grade 3, 8.4 % in grade 4, and 8.1 % in grade 5. The majority of participants had no prior experience with internet-based psychological help (93 %) and were not currently receiving psychological help (91 %). Additionally, 68 % reported no past psychological help, 86 % had no psychiatric diagnosis, and 95 % were not taking psychiatric medicine. For further information on the demographic characteristics and experiences of the participants, see Supplementary Table 1.

### 2.3 Measures

***Personal Information Form:*** A form developed by researchers to obtain information such as age, gender, educational status, and past psychological treatments of the participants.

***Internet-Based Intervention Preference Survey**:* The survey was designed to assess individual preferences regarding internet-based psychological interventions. This survey comprised 28 questions that gathered information on participants' past experiences with such interventions, their current utilisation of psychological services, and any history of psychiatric diagnoses or medication use. It inquired into the participants' daily internet usage and previous self-help experiences, exploring preferences for autonomous or therapist-supported intervention programs. Preferences for communication methods in therapist-assisted programs, including phone, face-to-face, or electronic messaging, were collected. The survey also determined preferences for the type of content (text or video) and the format (animation or real person). Technical preferences were addressed, such as the device type for accessing interventions, data protection concerns, and the use of introductory videos. Furthermore, the survey inquired about the desired structure of programs, encompassing total duration and session frequency, to understand participants' commitment capacities. It sought input on the preferred presentation of intervention modules, the inclusion of self-assessment tools, interactive response forms, and engagement reminders. Participants' preferences regarding character representation within the program and the inclusion of homework or relaxation exercises were also examined. It was created using fundamental studies (Apolinário-Hagen et al., 2018, Apolinário-Hagen et al., 2017; Berle et al., 2015), and tailored based on clinical observations to reflect the unique aspects of patient preferences and practical application. Participant responses, including frequencies and percentages, were presented in Supplementary Table 1.

***E-therapy attitudes measure (ETAM):*** The scale developed by Apolinário-Hagen et al. (2018) and adapted to Turkish by (Özer et al., In press) with the final version consisting of 16 items after one item was eliminated through confirmatory factor analysis to improve the scale's fit. The scale aims to assess individuals' attitudes towards internet-based interventions. The scale has two sub-dimensions: “perceived usefulness and helpfulness” and “advantage relative to face-to-face therapy”. Participants rated their agreement on a scale from 0 (‘strongly disagree’) to 4 (‘strongly agree’). The total possible scores on the ETAM range from 0 to 64, with higher scores indicating more positive attitudes towards e-therapy. The measurement tool's Cronbach's Alpha internal consistency coefficient was identified as 0.86, and it was 0.92 in this study.

***Digital Literacy Scale**:* The Digital Literacy Scale, developed by Ng (2012) and adapted to Turkish by Üstündağ et al. (2017), measures individuals' adaptability to new technological developments. Comprised of 10 items, this single-factor instrument utilises a 5-point Likert scale, allowing scores to range from 10 to 50. The scale's Cronbach Alpha reliability coefficient was found to be 0.86 in the original development and 0.88 in the current research. Higher scores on this scale indicate greater adaptability to technology usage, reflecting an individual's proficiency and comfort with engaging in digital environments.

***Patient Health Questionaire-9 (PHQ 9):*** The Patient Health Questionnaire-9 (PHQ-9) is a self-report instrument developed by Kroenke et al. (2001) and adapted into Turkish by Kroenke et al. (2001). It is designed to evaluate the level of depressive symptoms a person has experienced over the past two weeks. The questionnaire employs a 4-point Likert scale ranging from 0 (‘not at all’) to 3 (‘nearly every day’), with total scores varying from 0 to 27. Higher scores on the PHQ-9 indicate more severe depressive symptoms. The scoring categories are defined as mild (5–9), moderate (10–14), moderately severe (15–19), and severe depression (20–27). The internal consistency of the PHQ-9 is robust, with a coefficient reported as 0.84, suggesting that the instrument reliably measures the severity of depressive symptoms.

***Generalized Anxiety Disorder-7 (GAD-7):*** A measurement tool to evaluate participants' anxiety levels, developed by Spitzer et al. (2006) and adapted to Turkish by Konkan et al. (2013). The total scores obtained from the scale are cut-off points for mild, moderate, and severe anxiety at 5, 10, and 15, respectively. The Turkish adaptation study stated that individuals scoring eight and above should be investigated and confirmed for GAD diagnosis using other methods and that this can be used as a cut-off point. The GAD-7 test total score has a reported Cronbach's alpha value of 0.852. The Generalized Anxiety Disorder-7 (GAD-7) scale employs a 4-point Likert scoring system ranging from 0 (‘not at all’) to 3 (‘nearly every day’), allowing for total scores between 0 and 21. Higher scores denote increased anxiety symptoms.

### 2.4 Statistical analysis

Various statistical analyses were employed to examine the relationships among the study variables. Descriptive statistics, including measures of skewness and kurtosis, were used to assess the distribution of the variables. Pearson correlation analysis was conducted to explore the linear associations among the variables. Multiple regression analysis was then utilised to investigate the role of age, gender, digital literacy, anxiety, and depression as predictors of attitudes towards guided internet interventions. Assumptions underlying the regression analysis, such as multicollinearity, were carefully checked and met. All statistical analyses were performed using R studio (version 4.3.1). A *p*-value of 0.05 was established as the cut-off for statistical significance in all analyses conducted within this study.

## 3 Results

### 3.1 Descriptive statistics

The assumptions of normality were met for the variables under study. Independent variables included in the multiple regression analysis were not highly correlated with each other. We employed a standard deviation method from the mean of the digital literacy scores and defined ‘Low Digital Literacy’ as scores more than one standard deviation below the mean, ‘High Digital Literacy’ as scores more than one standard deviation above the mean, and ‘Medium Digital Literacy’ as scores within one standard deviation of the mean.

The analysis of digital literacy levels among the participants reveals that a significant majority of students (*N* = 169, 61.90 %) displayed medium digital literacy, indicating a proficiency that should enable them to engage effectively with internet-based interventions. However, a noteworthy proportion of the sample showed lower levels of digital literacy, with 17.95 % (*N* = 49) categorised as ‘Low’ and 20.15 % (*N* = 55) as ‘High.’ This distribution suggests that while most students possess sufficient digital skills for such interventions, a tailored approach might be necessary. For students with lower digital literacy levels, interventions could include additional support or introductory training to enhance their comfort and competence with digital tools.

### 3.2 Preferences in internet-based interventions

The survey data from Supplementary Table 1 revealed that a vast majority of students had not experienced internet-based psychological help in the past (93 %), with a similar proportion not currently receiving psychological help (91.2 %). Past psychological help was more prevalent, with 31.9 % of participants reporting such experience. Concerning psychiatric diagnostics and medication, most students had not been diagnosed (86.1 %) or were not taking psychiatric medications (94.9 %).

A significant finding was the high percentage of students (72.5 %) who sought information on their psychological symptoms. Regarding internet usage, the majority spent between 3 and 7 h daily (47.3 %), with a smaller group exceeding 7 h (18.7 %). In terms of self-help, most participants (63.4 %) were unfamiliar with self-help prior to the study, and a majority preferred therapist-assisted/support (64.1 %) over program-based interventions without therapist support (25.6 %). For those preferring therapist-assisted applications, face-to-face sessions were highly favored (62.6 %), and when considering content type, video (47.6 %) and specifically real-person videos (49.8 %) were the preferred formats.

Device usage leaned towards smartphones (57.5 %), and a substantial majority of participants valued data protection (88.3 %) and preferred the inclusion of an introduction video (83.9 %). When it came to program duration, the preferred mode was completion over extended periods rather than in a single day. For a program lasting 120 min, most preferred spreading it over 10 days (54.6 %), and for programs lasting 300 min, completion over 5 weeks was more popular (55.3 %).

In addition to the previously mentioned preferences, 37 % favored a more intensive schedule of 60 min daily for 5 consecutive days. The 10 Sessions Program saw varied responses, with 37.4 % preferring one session per day and 29.7 % opting for two sessions per week. Regarding time limitations for completion, a majority (60.8 %) preferred having a set time limit to complete the intervention. The most popular choice for the module duration in one sitting was 30–45 min (46.9 %), followed by 15 min (29.3 %). When it came to the presentation of intervention modules, a majority of students (54.2 %) preferred a new module every week, with 31.1 % favoring a daily module presentation. Only a small proportion (4.8 %) opted for a monthly module presentation.

The survey also indicated a strong preference for the inclusion of self-assessment questionnaires (85.3 %), interactive response forms (79.5 %), and regular reminders for intervention continuation, with once-a-week reminders being the most favored (40.3 %). Character identification within the program was also highly valued (75.1 %), as was the inclusion of homework (63.7 %) and relaxation exercises (78.4 %).

### 3.3 Prediction of attitudes towards guided internet interventions

The relationships between the main variables of interest, including age, digital literacy, anxiety, depression, and attitudes towards guided internet interventions, were explored using Pearson correlation analysis. This analysis provided insights into the linear associations among the variables, serving as a basis for the subsequent regression modelling. The detailed correlation coefficients, including statistical significance levels, are presented in Table 1.

**Table 1:** Means, standard deviations, and correlations with confidence intervals.

Variable	*M*	*SD*	1	2	3	4	5	6
1. Age	22.41	5.47						
2. Digital literacy	35.05	8.74	0.09					
3. Anxiety	10.13	5.77	−0.20^⁎⁎^	−0.11				
4. Depressive symptoms	12.80	6.63	−0.20^⁎⁎^	−0.17^⁎⁎^	0.76^⁎⁎^			
5. E-therapy attitudes - perceived usefulness and helpfulness	20.39	5.45	0.03	0.49^⁎⁎^	0.08	0.02		
6. E-therapy attitudes - advantage relative to face-to-face therapy	19.58	7.47	0.06	0.40^⁎⁎^	0.04	−0.03	0.68^⁎⁎^	
7. E-therapy attitudes – total score	39.97	11.85	0.05	0.48^⁎⁎^	0.06	−0.01	0.89^⁎⁎^	0.94^⁎⁎^

*Note. M* and *SD* are used to represent mean and standard deviation, respectively.

^⁎^*p* < .05.

^⁎⁎^*p* < .01.

### 3.4 Perceived usefulness and helpfulness

A multiple regression analysis was conducted to investigate the role of gender, age, digital literacy, anxiety, and depression as predictors of attitudes towards guided internet interventions in terms of perceived usefulness and helpfulness. The results of the regression analysis are summarised (see Table 2).

**Table 2:** Regression analysis on factors associated with attitudes towards internet-based intervention usefulness and helpfulness.

Parameter	*b*	95 % CI (*b*)	*t*	*df*	*p*	ß	95 % CI (ß)
(Intercept)	6.91⁎⁎	[2.98, 10.84]	3.46	267	0.001	0.00	[−0.10, 0.10]
Gender (Female)	0.68	[−0.61, 1.97]	1.03	267	0.302	0.06	[−0.05, 0.17]
Age	0.03	[−0.08, 0.14]	0.59	267	0.555	0.03	[−0.08, 0.14]
Digital Literacy	0.31⁎⁎	[0.25, 0.38]	9.45	267	< 0.001	0.51	[0.40, 0.61]
Anxiety	0.11	[−0.04, 0.26]	1.44	267	0.150	0.12	[−0.04, 0.28]
Depression	0.01⁎	[−0.12, 0.14]	0.13	267	0.894	0.01	[−0.15, 0.17]

*Note. R*^*2*^ = 0.262** 95 % CI[0.17,0.33]; *F* (5, 267) = 18.99, *p* = .000, A significant *b*-weight indicates the beta-weight and semi-partial correlation are also significant. *b* represents unstandardised regression weights. *Beta* indicates the standardised regression weights. *LL* and *UL* indicate the lower and upper limits of a confidence interval, respectively.

⁎ Indicates *p* < .05.

⁎⁎ Indicates *p* < .01.

The overall model was statistically significant, F(5, 267) = 18.99, *p* < .001, explaining approximately 26.23 % of the variance in Attitudes towards Guided Internet Interventions - Perceived Usefulness and Helpfulness subscale. Among the predictor variables, digital literacy emerged as the most significant predictor of perceived usefulness and helpfulness, with a positive relationship (β = 0.32, SE = 0.033, *t*(267) = 9.453, *p* < .001). The results suggest that an increase in digital literacy is associated with more favourable attitudes towards guided internet interventions in terms of perceived usefulness and helpfulness. The other predictor variables, including gender, age, anxiety, and depression, were not statistically significant predictors of this subscale (*p* > .05 for all).

In conclusion, the findings indicate that digital literacy is a crucial determinant of attitudes towards guided internet interventions in terms of perceived usefulness and helpfulness, while other variables in the model did not demonstrate a significant impact.

### 3.5 Perceived advantage relative to face-to-face therapy

A separate multiple regression analysis was conducted to examine the role of gender, age, digital literacy, anxiety, and depression as predictors of attitudes towards guided internet interventions in terms of perceived advantage relative to face-to-face therapy. The results are given in Table 3.

**Table 3:** Regression analysis on factors associated with attitudes towards internet-based intervention advantage relative to face-to-face therapy.

Parameter	*b*	95 % CI (*b*)	*t*	*df*	*p*	ß	95 % CI (ß)
(Intercept)	5.81⁎	[0.10, 11.51]	2.00	267	0.046	−0.00	[−0.11, 0.11]
Gender (Female)	−0.36	[−2.24, 1.52]	−0.37	267	0.710	−0.02	[−0.14, 0.10]
Age	0.04	[−0.11, 0.20]	0.55	267	0.581	0.03	[−0.08, 0.15]
Digital Literacy	0.34⁎⁎	[0.25, 0.44]	7.10	267	< 0.001	0.40	[0.29, 0.51]
Anxiety	0.19	[−0.03, 0.41]	1.67	267	0.096	0.14	[−0.03, 0.31]
Depression	−0.07	[−0.26, 0.12]	−0.72	267	0.470	−0.06	[−0.23, 0.11]

*Note. R*^*2*^ = 0.171** 95 % CI[0.08,0.24]; *F* (5, 267) = 11.01, *p* = .000, A significant *b*-weight indicates the beta-weight and semi-partial correlation are also significant. *b* represents unstandardised regression weights. *Beta* indicates the standardised regression weights. *LL* and *UL* indicate a confidence interval's lower and upper limits, respectively.

⁎ Indicates *p* < .05.

⁎⁎ Indicates *p* < .01.

The overall model was statistically significant, F(5, 267) = 11.01, *p* < .001, accounting for approximately 17.1 % of the variance in Attitudes towards Guided Internet Interventions - perceived advantage relative to face-to-face therapy subscale.

Among the predictors, digital literacy was found to be a significant positive predictor of attitudes towards guided internet interventions in terms of perceived advantage relative to face-to-face therapy (β = 0.34, SE = 0.048, *t*(267) = 7.095, *p* < .001), implying that greater digital literacy is associated with more favourable attitudes regarding the perceived advantage of guided internet interventions relative to face-to-face therapy.

Anxiety, though, demonstrated a marginally significant positive relationship with this subscale (β = 0.19, SE = 0.11, *t*(267) = 1.669, *p* = .0964). Other variables, including gender, age, and depression, did not reach statistical significance (*p* > .05 for all).

In conclusion, this analysis suggests that digital literacy plays a substantial role in shaping attitudes towards guided internet interventions concerning their perceived advantage over face-to-face therapy. The marginal significance of anxiety might also indicate a potential area for further exploration.

## 4 Discussion

### 4.1 Prediction of attitudes towards Internet-based interventions

To the best of our knowledge, this research is the first to assess the attitudes and preferences of university students in Turkey towards internet-based interventions. Studies related to internet-based interventions in Turkey are considerably limited. The initial objective of the study is to identify the relationship between university students' attitudes towards internet-based interventions and variables such as depression, anxiety, digital literacy, age, and gender. Consistent with previous research, it has been found that digital literacy is a positive predictor of attitudes towards internet-based interventions (Hernandez-Ramos et al., 2021; Wallin et al., 2016). Students with high digital literacy possess the necessary skills to use technology and have confidence in the digital environment. Consequently, they can better explore internet-based psychological support processes, distinguish correct information, and select reliable sources. Although university students are frequent internet users, low digital literacy is associated with negative attitudes towards internet-based interventions. This finding indicates the need for steps to enhance digital literacy skills to foster the adoption of internet-based interventions among university students.

This research shows that variables related to students, such as age and gender, are not associated with attitudes towards internet-based interventions. This research finding is consistent with a limited number of research results showing that gender is not associated with attitudes towards internet-based interventions (Apolinário-Hagen et al., 2018; Schröder et al., 2017). Although female participants are more prevalent in research concerning internet-based interventions, this does not stem from a negative attitude of males towards internet-based interventions but possibly from their reluctance to seek psychological help. Previous research has found significant relationships between age and attitudes towards internet-based interventions (Apolinário-Hagen et al., 2018). In this study, the participant group consists of university students who are in a similar developmental period, which might have prevented the emergence of this disparity.

According to another finding of the research, there is no relationship between the level of anxiety and depression and the attitude towards internet-based interventions. The findings of this research are consistent with the limited number of studies in the literature. Schröder et al. (2017) found in their research with individuals with depression that there was no significant difference between the attitudes towards internet-based interventions of individuals included in the research from non-clinical environments and those in a clinical setting. However, they noted a significant difference between participants in a clinical environment and those recruited from outside the clinic. This situation indicates that it arises from the presentation of psychological help rather than the level of depression. In this research, conditions such as 86 % of the participants not having received a psychiatric diagnosis and 68 % never having received psychological assistance may stem from being unaware of the differences in the provision of psychological help.

### 4.2 Preferences in internet-based interventions

According to the findings of this research, 97.1 % of students spend more than an hour a day on the internet using a smartphone/tablet/computer. Additionally, 73 % of the students state that they conduct research on the internet to obtain information about the mental distress they experience. Parallel to the findings, the literature also indicates that it serves as a significant resource while seeking help related to their mental health or obtaining information about symptoms related to mental disorders for young people (Wetterlin et al., 2014). However, a large majority of the students in the research (63.4 %) mentioned that they heard about internet-based interventions for the first time. Although this field is still developing in Turkey, the literature notes that there is low awareness and usage of Internet-Based Interventions (IBIs) (Apolinário-Hagen et al., 2019; Topooco et al., 2022). A lack of knowledge about internet-based interventions hinders their usage and adoption. In addition, the design features of internet-based interventions also affect the effectiveness of the intervention. According to the results of the meta-analysis, which included 46 studies (*n* = 16,632) with different design features, it was found that persuasive design in interventions for depression was positively associated with intervention results (McCall et al., 2021). There is a need for studies related to developing IBIs for university students in Turkey and announcing them.

According to the research findings, a significant portion of students prefer to use guided internet-based interventions (64.1 %), 25.6 % prefer unguided interventions, and 10.3 % indicated they would not use such interventions in any form. This finding corroborates research conclusions that highlight a preference among students for guided IBIs (Gericke et al., 2021; Topooco et al., 2022). While students prefer face-to-face expert support (62.6 %), there is no significant preference difference between support provided through SMS, email, or phone. This aligns with literature suggesting that students prefer face-to-face communication due to concerns about inadequately conveying their experiences, feelings, and thoughts through writing (Farrer et al., 2015). It also reinforces the literature-identified preference for more human support within IBIs (Fleischmann et al., 2018; Peynenburg et al., 2020; Salamanca-Sanabria et al., 2023).

While the current study indicates a preference among students for guided internet-based interventions (IBIs), it is important to note that these findings are based solely on students' perceptions rather than direct engagement with IBIs. Furthermore, it is worth considering the results of the meta-analysis by Harrer et al. (2019), which did not find guidance to be a significant factor in treatment outcomes for student populations. This suggests that while economic and workforce efficiencies are clear advantages of unguided IBIs, the impact of the guidance on intervention effectiveness, particularly within student populations, may require further investigation to understand its role and relevance fully.

The majority of students prefer to use internet-based intervention (IBI) through mobile devices (57.5 %) over using them on computers (27.1 %). Although this finding mirrors some literature (Schouten et al., 2023), earlier studies have found a preference for using laptop or desktop devices over mobile applications (Batterham and Calear, 2017). The discrepancy may be due to the variations in the age groups of the research participants and the increasing user-friendliness and prevalence of mobile applications. While the medium through which the IBI is offered is crucial, another vital aspect is the provision of information. A considerable proportion of students prefer having an informational video before using an IBI (83.9 %). Informative content reduces the difficulties associated with using IBIs and enhances their acceptance by increasing perceived ease of use (Holtz et al., 2020; Russell et al., 2018). When developing internet-based interventions, it should be taken into account that students require detailed information. Moreover, how their personal information will be protected is crucial for students, with 88.3 % desiring information on this matter. Concerns about data security among students are highlighted in the literature (Chan et al., 2016b; Farrer et al., 2015; Gericke et al., 2021; Levin et al., 2018). Similarly, (Musiat et al., 2014) underscored that perceived trustworthiness profoundly influences the use of IBIs. It is imperative for IBI developers to be more explanatory regarding this matter.

A large majority of students prefer having a time limit to complete the interventions within the IBI application (60.8 %). Similarly, they indicated that unpredictably long sessions disrupt the regular completion of the module (Fleischmann et al., 2018). Reminders are utilised to encourage completion and for guidance purposes (Harrer et al., 2019). Most students prefer weekly reminders (40.3 %), with daily (23.8 %) and thrice-a-week reminders (23.4 %) being similarly preferred. While 2.6 % of students did not want reminders, 9.9 % had no strong opinion. These results demonstrate that university students feel the necessity for time limits and reminders in the process of using internet-based interventions.

In this study, a large proportion of students indicated a preference for scales/surveys for self-evaluation in the IBI application (85.3 %). Measurement tools in IBIs are beneficial for fostering mental health awareness among users and enabling them to track and evaluate their progress over time. However, they should be informed about the limitations of the evaluations derived from the measurement results and how to interpret these results. Additionally, they prefer having a character within the intervention application with whom they can identify (75.1 %). The literature points out that students find IBIs more engaging through story-based designs (Salamanca-Sanabria et al., 2023). An avatar increases self-representation through a virtual character and enhances the ability to control this character.

According to another finding of the research, students prefer the information in the content to be presented through videos rather than in writing, and they prefer real people to be featured in these videos. Visual and auditory learning aids them in understanding the information more effectively. Videos provide a more visual and emotional experience than texts, helping students grasp and contextualise the content more easily. Having real people in the videos can make the content more meaningful by allowing students to empathise and learn in a human context. Additionally, students prefer having interactive forms where they can write their responses (79.5 %), with 6.2 % opposing this. This finding does not align with the literature, where participants involved in internet-based interventions (IBIs) are reluctant to write and prefer fewer writing activities (Fleischmann et al., 2018). The discrepancy here might stem from the fact that the majority of the study group has not experienced this before. Students prefer to have auditory relaxation/relaxation exercises in the IBI application (78.4 %). Auditory exercises are noted to be more beneficial in IBI interventions than writing activities (Lantto, 2021). Alongside this, the majority of students prefer having tasks/assignments to transfer the interventions/information from the IBI application into their daily lives (63.7 %). It is noted that young individuals in the group undergoing the computerised behavioural activation program for depression experienced concerns regarding homework and desired it to be at a minimal level (Tindall et al., 2021). However, it is also noted that there is a connection between adherence to homework in cognitive behavioural therapy (CBT) applications and clinical improvements (Simons et al., 2012).

It can be inferred from these findings that creating a more interactive and multidimensional learning environment in IBIs, which includes visual and auditory elements, real people, and practical tasks, can potentially make the intervention more effective and appealing to students. However, the challenge remains to balance the level of interaction and task assignments to avoid overwhelming the students while still encouraging active participation and engagement in the intervention process. It is essential to carefully design the interventions, keeping in mind the preferences of the students, and to align them with evidence-based practices from existing literature to enhance the effectiveness of IBIs. Providing clear guidance and instructions is also pivotal to helping students integrate the knowledge and skills acquired through the interventions into their daily lives. Integrating real-life tasks and assignments can foster a more hands-on approach to learning and facilitate the transfer of skills and knowledge from the virtual environment to the real world. The diverse preferences among students suggest a need for flexible and customisable intervention designs that cater to different learning styles and preferences, promoting inclusivity and effectiveness in IBIs.

Students prefer to use a 120-minute program daily over a span of 10 days rather than completing it in a single day (54.6 %). Similarly, rather than using a 300-minute program for 60 min consecutively over 5 days (37 %), it is preferred to use it for 60 min weekly over a total of 5 weeks (45.1 %). Students prefer completing the program over a more extended period in small content portions rather than finishing the entire content quickly through intensive study. However, if the program contains longer content, they prefer having a longer time between the sections. Additionally, they prefer to complete the entire program in 5 weeks rather than 15 weeks. These findings are consistent with other research conducted on the duration and frequency related to Internet-based interventions (IBIs) (Batterham and Calear, 2017). Moreover, it has been indicated that interventions lasting between 5 and 8 weeks are more effective than those <4 weeks and >9 weeks (Heber et al., 2017; Richards and Richardson, 2012).

In the framework of Internet-based intervention applications, they prefer interventions to be structured in such a way that a new module can be completed every week (54.2 %). Their other preferences are for the modules to be completed daily (31.1 %) and, least preferred, to be completed monthly (4.8 %). They also prefer for a module to be completed with a work duration of 30–45 min (46.9 %), followed by a 15-minute session (29.3 %) and a 45–60 min session (9.9 %). This aligns with findings in the literature that suggest a duration of 30–45 min is suitable (Tindall et al., 2021). IBIs offer students flexibility in terms of time and space. Therefore, students generally prefer to plan sessions according to their schedules. Several short sessions a week allows students to focus on their classes and other responsibilities while simultaneously receiving psychological support.

It is essential to underline that these preferences point towards the necessity of offering flexible modules that can be adapted to individual schedules and learning paces. This would enable students to integrate the intervention program smoothly into their daily routines, thus possibly enhancing adherence and effectiveness of the IBIs. It highlights the need to consider individual preferences in the design of IBIs to foster a user-friendly environment that encourages consistent participation and engagement while aligning with proven effective intervention durations from existing research. It may be worthwhile to explore a modular approach that allows for customisation in terms of duration and frequency to cater to a diverse range of preferences and enhance the efficacy and accessibility of IBIs.

### 4.3 Limitations

The participant group was limited to students at a state university in the Black Sea region of Turkey. This demographic homogeneity may decrease the external validity of the findings. The university's specific characteristics, such as its geographical location and student population, might not represent the diverse experiences and backgrounds of university students across Turkey. Future research could include a more varied sample from different regions to increase generalizability.

The study's correlational design explores relationships between variables but cannot establish cause-and-effect relationships. This limitation means that while associations were observed, these relationships' directionality and underlying mechanisms remain uncertain. The use of a questionnaire form to ascertain student preferences might not capture the depth and complexity of their attitudes towards IBIs. The data collected reflects self-reported preferences, which could be influenced by students' limited exposure to or understanding of internet-based interventions. Future studies could employ more diverse measurement tools, such as qualitative interviews or observational methods, to obtain a richer and more nuanced understanding of student preferences.

The novelty of internet-based interventions in Turkey implies that participants might lack comprehensive knowledge about these interventions, potentially leading to hypothetical interpretation of preferences-related issues. This lack of familiarity could have influenced students to express preferences based on limited understanding rather than informed experience. The preferences reported may, therefore, more accurately reflect perceptions of IBIs or assumptions about digital interventions (Soucy et al., 2016).

This study utilised a general digital literacy scale to measure digital literacy. However, other studies have employed the Digital Health Literacy Instrument (DHLI) (van der Vaart and Drossaert, 2017) to specifically assess competencies related to internet interventions. The Turkish version of this instrument was only recently published (Çetin and Gümüş, 2023) and was not available during our study's timeframe. Future research in this area could benefit from using the DHLI to measure digital health literacy more accurately, which may directly impact attitudes and preferences towards internet-based interventions.

### 4.4 Strengths of the study

In addition to its limitations, the current study holds several strengths. One of the first to assess university students' attitudes and preferences towards internet-based interventions in Turkey, provides a unique contribution to the field. Using a diverse array of validated instruments, alongside the specially developed Internet-Based Intervention Preference Survey, allows for a comprehensive evaluation of students' attitudes, digital literacy, and mental health status. Moreover, the study contributes significantly to the literature by identifying digital literacy as a critical predictor of attitudes towards internet-based interventions. This highlights the importance of enhancing digital literacy to foster the adoption and effectiveness of such interventions among university students, a perspective not extensively explored in previous research. The study also addresses a significant gap by providing insights into students' specific preferences for the features of internet-based interventions. The detailed analysis of preferences related to intervention structure, content, and delivery format offers valuable guidance for developers of these interventions, ensuring they are tailored to university students' unique needs and expectations.

## 5 Conclusion

In conclusion, this study identified the attitudes of university students in Turkey towards IBIs and their preferences concerning the components, duration, frequency, presentation style, content, and method of IBIs. Digital literacy predicts positive attitudes towards internet-based interventions. Demographic variables such as age, gender, and levels of depression and anxiety are unrelated to the attitudes towards IBIs. A large majority of students in Turkey are unaware of IBIs. Students prefer IBIs with more human interaction, including face-to-face guidance support and video content featuring people. They seek information on how to use IBIs and how their data will be protected. They neither prefer interventions that last for weeks nor those completed intensively over consecutive days; they favour completing one or two sections per week. Students prefer IBIs that feature scales allowing self-assessment, a character with whom they can identify, voice relaxation exercises, activity tasks that they can implement in their daily lives, and weekly reminders throughout this process. Developers of IBIs targeting university students can take these preferences into consideration. Considering these preferences can enhance the benefits students receive and increase their engagement with IBIs.

Implementing IBIs based on the identified preferences can foster a more user-friendly environment, encouraging participation and engagement. Moreover, aligning IBIs with the students' preferences could potentially enhance their effectiveness, thereby promoting greater benefits and fostering increased student commitment. This approach ensures that IBIs are not only accessible but also adequately suited to meet the diverse needs and preferences of university students, thereby fostering an environment conducive to positive outcomes. Future research in this area might explore the optimisation of IBIs through the incorporation of user preferences to enhance both adherence and effectiveness. It remains critical to continue researching this area to adapt further and optimise IBIs to cater to the specific needs and preferences of university students in Turkey.

The following is the supplementary data related to this article.

Supplementary Table 1 Sample characteristic (N = 273). Supplementary Table 1

## Declaration of competing interest

The authors declare that they have no known competing financial interests or personal relationships that could have appeared to influence the work reported in this paper.

## References

[bb0005] Akhtar P., Ma L., Waqas A., Naveed S., Li Y., Rahman A., Wang Y. (2020). Prevalence of depression among university students in low and middle income countries (LMICs): a systematic review and meta-analysis. J. Affect. Disord..

[bb0010] Akin-Sari B., Inozu M., Haciomeroglu A.B., Cekci B.C., Uzumcu E., Doron G. (2022). Cognitive training via a Mobile application to reduce obsessive-compulsive-related distress and cognitions during the COVID-19 outbreaks: a randomized controlled trial using a subclinical cohort. Behav. Ther..

[bb0015] Akin-Sari B., Inozu M., Haciomeroglu A.B., Trak E., Tufan D., Doron G. (2022). Cognitive training using a mobile app as a coping tool against COVID-19 distress: a crossover randomized controlled trial. J. Affect. Disord..

[bb0020] Amanvermez Y., Karyotaki E., Cuijpers P., Salemink E., Spinhoven P., Struijs S., de Wit L.M. (2021). Feasibility and acceptability of a guided internet-based stress management intervention for university students with high levels of stress: protocol for an open trial. Internet Interv..

[bb0025] Andersson G., Titov N. (2014). Advantages and limitations of internet-based interventions for common mental disorders. World Psychiatry.

[bb0030] APA Presidential Task Force on Evidence-Based Practice (2006). Evidence-based practice in psychology. Am. Psychol..

[bb1000] Apolinário-Hagen J., Vehreschild V., Alkoudmani R.M. (2017). Current views and perspectives on e-mental health: An exploratory survey study for understanding public attitudes toward internet-based psychotherapy in Germany. JMIR Mental Health.

[bb0035] Apolinário-Hagen J., Harrer M., Kählke F., Fritsche L., Salewski C., Ebert D.D. (2018). Public attitudes toward guided internet-based therapies: web-based survey study. JMIR Mental Health.

[bb0040] Apolinário-Hagen J., Hennemann S., Fritsche L., Drüge M., Breil B. (2019). Determinant factors of public acceptance of stress management apps: survey study. JMIR Mental Health.

[bb0045] Apolinário-Hagen J., Harrer M., Salewski C., Lehr D., Ebert D.D. (2022). Akzeptanz und Nutzung von E-Mental-Health-Angeboten unter Studierenden. Prävention und Gesundheitsförderung..

[bb0050] Auerbach R.P., Alonso J., Axinn W.G., Cuijpers P., Ebert D.D., Green J.G., Hwang I., Kessler R.C., Liu H., Mortier P., Nock M.K., Pinder-Amaker S., Sampson N.A., Aguilar-Gaxiola S., Al-Hamzawi A., Andrade L.H., Benjet C., Caldas-de-Almeida J.M., Demyttenaere K., Bruffaerts R. (2016). Mental disorders among college students in the World Health Organization world mental health surveys. Psychol. Med..

[bb0055] Auerbach Randy P., Mortier P., Bruffaerts R., Alonso J., Benjet C., Cuijpers P., Demyttenaere K., Ebert D.D., Green J.G., Hasking P., Murray E., Nock M.K., Pinder-Amaker S., Sampson N.A., Stein D.J., Vilagut G., Zaslavsky A.M., Kessler R.C. (2018). WHO world mental health surveys international college student project: prevalence and distribution of mental disorders. J. Abnorm. Psychol..

[bb0060] Batterham P.J., Calear A.L. (2017). Preferences for internet-based mental health interventions in an adult online sample: findings from an online community survey. JMIR Mental Health.

[bb0065] Bauer S., Moessner M., Wolf M., Haug S., Kordy H. (2009). ES[S]PRIT – an internet-based programme for the prevention and early intervention of eating disorders in college students. Br. J. Guid. Counsel..

[bb1005] Berle D., Starcevic V., Milicevic D., Hannan A., Dale E., Brakoulias V., Viswasam K. (2015). Do patients prefer face-to-face or internet-based therapy?. Psychotherapy and Psychosomatics.

[bb0070] Blanco C., Okuda M., Wright C., Hasin D.S., Grant B.F., Liu S.-M., Olfson M. (2008). Mental health of college students and their non–college-attending peers: results from the National Epidemiologic Study on alcohol and related conditions. Arch. Gen. Psychiatry.

[bb0075] Bruffaerts R., Mortier P., Auerbach R.P., Alonso J., Hermosillo De la Torre A.E., Cuijpers P., Demyttenaere K., Ebert D.D., Green J.G., Hasking P., Stein D.J., Ennis E., Nock M.K., Pinder-Amaker S., Sampson N.A., Vilagut G., Zaslavsky A.M., Kessler R.C., Collaborators W.W.-I. (2019). Lifetime and 12-month treatment for mental disorders and suicidal thoughts and behaviors among first year college students. Int. J. Methods Psychiatr. Res..

[bb0080] Çetin M., Gümüş R. (2023). Research into the relationship between digital health literacy and healthy lifestyle behaviors: an intergenerational comparison. Front. Public Health.

[bb0085] Chan J.K., Farrer L.M., Gulliver A., Bennett K., Griffiths K.M. (2016). University students’ views on the perceived benefits and drawbacks of seeking help for mental health problems on the internet: a qualitative study. JMIR Hum. Factors.

[bb0090] Chan J.K., Farrer L.M., Gulliver A., Bennett K., Griffiths K.M. (2016). University students’ views on the perceived benefits and drawbacks of seeking help for mental health problems on the internet: a qualitative study. JMIR Hum. Factors.

[bb0095] Chiu T., Marziali E., Colantonio A., Carswell A., Gruneir M., Tang M., Eysenbach G. (2009). Internet-based caregiver support for Chinese Canadians taking care of a family member with Alzheimer disease and related dementia. Can. J. Aging/La Revue Canadienne Du Vieillissement.

[bb0100] Cuijpers P., Schuurmans J. (2007). Self-help interventions for anxiety disorders: an overview. Curr. Psychiatry Rep..

[bb0105] Czyz E.K., Horwitz A.G., Eisenberg D., Kramer A., King C.A. (2013). Self-reported barriers to professional help seeking among college students at elevated risk for suicide. J. Am. Coll. Health.

[bb0110] Dorow M., Löbner M., Pabst A., Stein J., Riedel-Heller S.G. (2018). Preferences for depression treatment including internet-based interventions: results from a large sample of primary care patients. Front. Psychiatry.

[bb0115] Ebert D.D., Mortier P., Kaehlke F., Bruffaerts R., Baumeister H., Auerbach R.P., Alonso J., Vilagut G., Martínez K.U., Lochner C., Cuijpers P., Kuechler A.-M., Green J., Hasking P., Lapsley C., Sampson N.A., Kessler R.C., Collaborators, O. behalf of the W. W. M. H.-I. C. S. I (2019). Barriers of mental health treatment utilization among first-year college students: first cross-national results from the WHO world mental health international college student initiative. Int. J. Methods Psychiatr. Res..

[bb0120] Eisenberg D., Downs M.F., Golberstein E., Zivin K. (2009). Stigma and help seeking for mental health among college students. Med. Care Res. Rev..

[bb0125] Farrer L., Gulliver A., Chan J.K., Bennett K., Griffiths K.M. (2015). A virtual mental health Clinic for University Students: a qualitative study of end-user service needs and priorities. JMIR Mental Health.

[bb0130] Fleischmann R.J., Harrer M., Zarski A.-C., Baumeister H., Lehr D., Ebert D.D. (2018). Patients’ experiences in a guided internet- and app-based stress intervention for college students: a qualitative study. Internet Interv..

[bb0135] Gao L., Xie Y., Jia C., Wang W. (2020). Prevalence of depression among Chinese university students: a systematic review and meta-analysis. Sci. Rep..

[bb0140] Garlow S.J., Rosenberg J., Moore J.D., Haas A.P., Koestner B., Hendin H., Nemeroff C.B. (2008). Depression, desperation, and suicidal ideation in college students: results from the American Foundation for Suicide Prevention College screening project at Emory University. Depress. Anxiety.

[bb0145] Gericke F., Ebert D.D., Breet E., Auerbach R.P., Bantjes J. (2021). A qualitative study of university students’ experience of internet-based CBT for depression. Couns. Psychother. Res..

[bb0150] Goates-Jones M., Hill C.E. (2008). Treatment preference, treatment-preference match, and psychotherapist credibility: influence on session outcome and preference shift. Psychotherapy (Chic.).

[bb0155] Göcek-Yorulmaz E. (2020).

[bb0160] Harrer M., Adam S.H., Fleischmann R.J., Baumeister H., Auerbach R., Bruffaerts R., Cuijpers P., Kessler R.C., Berking M., Lehr D., Ebert D.D. (2018). Effectiveness of an internet- and app-based intervention for college students with elevated stress: randomized controlled trial. J. Med. Internet Res..

[bb0165] Harrer M., Adam S.H., Baumeister H., Cuijpers P., Karyotaki E., Auerbach R.P., Kessler R.C., Bruffaerts R., Berking M., Ebert D.D. (2019). Internet interventions for mental health in university students: a systematic review and meta-analysis. Int. J. Methods Psychiatr. Res..

[bb0170] Heber E., Ebert D.D., Lehr D., Cuijpers P., Berking M., Nobis S., Riper H. (2017). The benefit of web- and computer-based interventions for stress: a systematic review and meta-analysis. J. Med. Internet Res..

[bb0175] Hernandez-Ramos R., Aguilera A., Garcia F., Miramontes-Gomez J., Pathak L.E., Figueroa C.A., Lyles C.R. (2021). Conducting internet-based visits for onboarding populations with limited digital literacy to an mHealth intervention: development of a patient-centered approach. JMIR Formative Res..

[bb1010] Holtz B.E., McCarroll A.M., Mitchell K.M. (2020). Perceptions and Attitudes Toward a Mobile Phone App for Mental Health for College Students: Qualitative Focus Group Study. JMIR Formative Research.

[bb0180] Hunt J., Eisenberg D. (2010). Mental health problems and help-seeking behavior among college students. J. Adolesc. Health.

[bb0185] Hustad J.T.P., Barnett N.P., Borsari B., Jackson K.M. (2010). Web-based alcohol prevention for incoming college students: a randomized controlled trial. Addict. Behav..

[bb0190] Jenkins P.E., Ducker I., Gooding R., James M., Rutter-Eley E. (2021). Anxiety and depression in a sample of UK college students: a study of prevalence, comorbidity, and quality of life. J. Am. Coll. Health.

[bb0195] Kählke F., Berger T., Schulz A., Baumeister H., Berking M., Auerbach R.P., Bruffaerts R., Cuijpers P., Kessler R.C., Ebert D.D. (2019). Efficacy of an unguided internet-based self-help intervention for social anxiety disorder in university students: a randomized controlled trial. Int. J. Methods Psychiatr. Res..

[bb0200] Kessler R.C., Amminger G.P., Aguilar-Gaxiola S., Alonso J., Lee S., Ustün T.B. (2007). Age of onset of mental disorders: a review of recent literature. Curr. Opin. Psychiatry.

[bb0205] Konkan R., Şenormancı Ö., Güçlü O., Aydın E., Sungur M.Z. (2013). Yaygın Anksiyete Bozukluğu-7 (YAB-7) Testi Türkçe Uyarlaması. Geçerlik ve Güvenirliği..

[bb0210] Kontos E., Blake K.D., Chou W.-Y.S., Prestin A. (2014). Predictors of eHealth usage: insights on the digital divide from the health information National Trends Survey 2012. J. Med. Internet Res..

[bb0215] Kroenke K., Spitzer R.L., Williams J.B. (2001). The PHQ-9: validity of a brief depression severity measure. J. Gen. Intern. Med..

[bb0220] Kypri K., Hallett J., Howat P., McManus A., Maycock B., Bowe S., Horton N.J. (2009). Randomized controlled trial of proactive web-based alcohol screening and brief intervention for university students. Arch. Intern. Med..

[bb0225] Lantto K. (2021). An online guided ACT intervention for students: what are the student experiences, and do they differ depending on anxiety level?. https://jyx.jyu.fi/handle/123456789/77009.

[bb0230] Lattie E.G., Adkins E.C., Winquist N., Stiles-Shields C., Wafford Q.E., Graham A.K. (2019). Digital mental health interventions for depression, anxiety, and enhancement of psychological well-being among college students: systematic review. J. Med. Internet Res..

[bb0235] Levin M.E., Hayes S.C., Pistorello J., Seeley J.R. (2016). Web-based self-help for preventing mental health problems in universities: comparing acceptance and commitment training to mental health education. J. Clin. Psychol..

[bb0240] Levin M.E., Stocke K., Pierce B., Levin C. (2018). Do College students use online self-help? A survey of intentions and use of mental health resources. J. Coll. Stud. Psychother..

[bb0245] Linardon J., Shatte A., Tepper H., Fuller-Tyszkiewicz M. (2020). A survey study of attitudes toward, and preferences for, e-therapy interventions for eating disorder psychopathology. Int. J. Eat. Disord..

[bb0250] Linardon J., Messer M., Lee S., Rosato J. (2021). Perspectives of e-health interventions for treating and preventing eating disorders: descriptive study of perceived advantages and barriers, help-seeking intentions, and preferred functionality. Eating Weight Disord. Stud. Anorexia Bulimia Obesity.

[bb0255] Marsh C.N., Wilcoxon S.A. (2015). Underutilization of mental health services among college students: an examination of system-related barriers. J. Coll. Stud. Psychother..

[bb0260] McCall H.C., Richardson C.G., Helgadottir F.D., Chen F.S. (2018). Evaluating a web-based social anxiety intervention among university students: randomized controlled trial. J. Med. Internet Res..

[bb0265] McCall H.C., Hadjistavropoulos H.D., Sundström C.R.F. (2021). Exploring the role of persuasive design in unguided internet-delivered cognitive behavioral therapy for depression and anxiety among adults: systematic review, meta-analysis, and meta-regression. J. Med. Internet Res..

[bb0270] McCloud T., Jones R., Lewis G., Bell V., Tsakanikos E. (2020). Effectiveness of a mobile app intervention for anxiety and depression symptoms in university students: randomized controlled trial. JMIR Mhealth Uhealth.

[bb0275] Musiat P., Conrod P., Treasure J., Tylee A., Williams C., Schmidt U. (2014). Targeted prevention of common mental health disorders in university students: randomised controlled trial of a transdiagnostic trait-focused web-based intervention. PloS One.

[bb0280] Newman M.G., Kanuri N., Rackoff G.N., Jacobson N.C., Bell M.J., Taylor C.B. (2021). A randomized controlled feasibility trial of internet-delivered guided self-help for generalized anxiety disorder (GAD) among university students in India. Psychotherapy.

[bb0285] Ng W. (2012). Can we teach digital natives digital literacy?. Comput. Educ..

[bb0290] Özer Ö., Ceyhan A.A., Struijs S.Y. (2023). User profile of an online cognitive behavioral therapy self-help platform in Turkey. Curr. Psychol..

[bb0295] Özer, Ö., Köksal, B., & Altinok, A. (In press). Turkish adaptation of E-therapy attitudes measure: validity and reliability study. Turkish J. Clin. Psychol. doi:10.57127/kpd.26024438.1241899.

[bb0300] Peynenburg V.A., Mehta S., Hadjistavropoulos H.D. (2020). Postsecondary student perceptions and preferences for the treatment of depression and anxiety: comparison of internet-delivered cognitive behaviour therapy to face-to-face cognitive behaviour therapy and medication. Can. J. Behav. Sci./Revue Canadienne Des Sciences Du Comportement.

[bb0305] Punton G., Dodd A.L., McNeill A. (2022). ‘You’re on the waiting list’: an interpretive phenomenological analysis of young adults’ experiences of waiting lists within mental health services in the UK. PloS One.

[bb0310] Richards D., Richardson T. (2012). Computer-based psychological treatments for depression: a systematic review and meta-analysis. Clin. Psychol. Rev..

[bb2000] Russell E., Lloyd-Houldey A., Memon A., Yarker J. (2018). Factors Influencing Uptake and Use of a New Health Information App for Young People. Journal of Technology in Human Services.

[bb0315] Salamanca-Sanabria A., Jabir A.I., Lin X., Alattas A., Kocaballi A.B., Lee J., Kowatsch T., Car L.T. (2023). Exploring the perceptions of mHealth interventions for the prevention of common mental disorders in university students in Singapore: qualitative study. J. Med. Internet Res..

[bb0325] Schouten M.J.E., Derksen M.E., Dekker J.J.M., Goudriaan A.E., Blankers M. (2023). Preferences of young adults on the development of a new digital add-on alcohol intervention for depression treatment: a qualitative study. Internet Interv..

[bb0330] Schröder J., Berger T., Meyer B., Lutz W., Hautzinger M., Späth C., Eichenberg C., Klein J.P., Moritz S. (2017). Attitudes towards internet interventions among psychotherapists and individuals with mild to moderate depression symptoms. Cogn. Ther. Res..

[bb0335] Simons A.D., Marti C.N., Rohde P., Lewis C.C., Curry J., March J. (2012). Does homework “matter” in cognitive behavioral therapy for adolescent depression?. J. Cogn. Psychother..

[bb0340] Soucy J.N., Owens V.A.M., Hadjistavropoulos H.D., Dirkse D.A., Dear B.F. (2016). Educating patients about internet-delivered cognitive behaviour therapy: perceptions among treatment seekers and non-treatment seekers before and after viewing an educational video. Internet Interv..

[bb0345] Spitzer R.L., Kroenke K., Williams J.B.W., Löwe B. (2006). A brief measure for assessing generalized anxiety disorder: the GAD-7. Arch. Intern. Med..

[bb0350] Sun S., Lin D., Goldberg S., Shen Z., Chen P., Qiao S., Brewer J., Loucks E., Operario D. (2022). A mindfulness-based mobile health (mHealth) intervention among psychologically distressed university students in quarantine during the COVID-19 pandemic: a randomized controlled trial. J. Couns. Psychol..

[bb0355] Swift J.K., Callahan J.L. (2009). The impact of client treatment preferences on outcome: a meta-analysis. J. Clin. Psychol..

[bb0360] Swift J.K., Callahan J.L., Cooper M., Parkin S.R. (2018). The impact of accommodating client preference in psychotherapy: a meta-analysis: SWIFT et al. J. Clin. Psychol..

[bb0365] Tindall L., Toner P., Mikocka-Walus A., Wright B. (2021). Perceptions of and opinions on a computerized behavioral activation program for the treatment of depression in young people: thematic analysis. J. Med. Internet Res..

[bb0370] Topooco N., Fowler L.A., Fitzsimmons-Craft E.E., DePietro B., Vázquez M.M., Firebaugh M.-L., Ceglarek P., Monterubio G., Newman M.G., Eisenberg D., Wilfley D.E., Taylor C.B. (2022). Digital interventions to address mental health needs in colleges: perspectives of student stakeholders. Internet Interv..

[bb0375] Üstündağ M.T., Güneş E., Bahçivan E. (2017). Dijital Okuryazarlık Ölçeğinin Türkçeye Uyarlanması ve Fen Bilgisi Öğretmen Adaylarının Dijital Okuryazarlık Durumları. J. Educ. Futur..

[bb0380] van der Vaart R., Drossaert C. (2017). Development of the digital health literacy instrument: measuring a broad Spectrum of health 1.0 and health 2.0 skills. J. Med. Internet Res..

[bb0385] Wallin E.E.K., Mattsson S., Olsson E.M.G. (2016). The preference for internet-based psychological interventions by individuals without past or current use of mental health treatment delivered online: a survey study with mixed-methods analysis. JMIR Mental Health.

[bb0390] Wetterlin F.M., Mar M.Y., Neilson E.K., Werker G.R., Krausz M. (2014). eMental health experiences and expectations: a survey of youths’ web-based resource preferences in Canada. J. Med. Internet Res..

