# Supplementary material for: Understanding university students' attitudes and preferences for internet-based mental health interventions
Source: Internet Interv. 2024 Feb 6;35:100722. doi: 10.1016/j.invent.2024.100722 (PMC10864831; doi:10.1016/j.invent.2024.100722)
Supplement: Supplementary Table 1 — Sample characteristic (N = 273). [file mmc1.docx]

Supplementary Table 1

*Sample Characteristic (N = 273)*

| Characteristic | N = 273^1^ |
| --- | --- |
| ***Demographics*** |  |
| Age (years) | 22.41 (5.47) |
| Gender |  |
| Female | 187 (68%) |
| Male | 86 (32%) |
| Grade (1-5, years) |  |
| 1^st^ Year Students | 110 (40%) |
| 2^nd^ Year Students | 78 (29%) |
| 3^rd^ Year Students | 40 (15%) |
| 4^th^ Year Students | 23 (8.4%) |
| 5^th^ Year Students | 22 (8.1%) |
| ***Psychological/Psychiatric Help and Diagnostics*** |  |
| Interned-based psychological help experience in the past |  |
| No | 254 (93%) |
| Yes | 19 (7.0%) |
| Psychological help - current |  |
| No | 249 (91.2%) |
| Yes | 24 (8.8%) |
| Psychological help - past |  |
| No | 186 (68.1%) |
| Yes | 87 (31.9%) |
| Having a psychiatric diagnostic |  |
| No | 235 (86.1%) |
| Yes | 38 (13.9%) |
| Taking a psychiatric medicine |  |
| No | 259 (94.9%) |
| Yes | 14 (5.1%) |
| Searching for information on their psychological symptoms |  |
| No | 75 (27.5%) |
| Yes | 198 (72.5%) |
| ***Internet Usage*** |  |
| Daily time spent on the internet (phone/computer/tablet etc.) |  |
| Less than one hour | 8 (2.9%) |
| 1-3 hours | 85 (31.1%) |
| 3-7 hours | 129 (47.3%) |
| More than 7 hours | 51 (18.7%) |
| ***Self-help*** |  |
| Self-help experience |  |
| I heard it for the first time | 173 (63.4%) |
| I knew but did not use it. | 77 (28.2%) |
| Used it before | 23 (8.4%) |
| Self-help forms |  |
| I do not prefer either way | 28 (10.3%) |
| Only program based, without therapist assistance/support | 70 (25.6%) |
| Prefer therapist-assisted/supported | 175 (64.1%) |
| if the application is therapist-assisted, |  |
| I do not have a specific preference | 39 (14.3%) |
| I prefer short calls by phone | 28 (10.3%) |
| I prefer short sessions face to face | 171 (62.6%) |
| I prefer SMS/e-mail | 35 (12.8%) |
| ***Preference about content, duration, frequency and method*** |  |
| Content preference |  |
| No preference | 66 (24.2%) |
| Text | 77 (28.2%) |
| Video | 130 (47.6%) |
| Video preference |  |
| No preference | 66 (24.2%) |
| Animation | 71 (26%) |
| Real person | 136 (49.8%) |
| Device preference |  |
| No preference | 42 (15.4%) |
| PC | 74 (27.1%) |
| Phone | 157 (57.5%) |
| Data protection information/declaration preference |  |
| No preference | 25 (9.2%) |
| No | 7 (2.6%) |
| Yes | 241 (88.3%) |
| Introduction video |  |
| No preference | 28 (10.2%) |
| No | 16 (5.9%) |
| Yes | 229 (83.9%) |
| Internet-Based Intervention Program lasting 120 mins |  |
| No preference | 61 (22.3%) |
| Completion in a day, 120 mins in a day | 63 (23.1%) |
| Completion in 10 days, 12 mins in a day | 149 (54.6%) |
| Internet-Based Intervention Program lasting 300 mins |  |
| No preference | 45 (16.5%) |
| Completion in 15 weeks, 20 mins per week | 77 (28.2%) |
| Completion in 5 weeks, 60 mins per week | 151 (55.3%) |
| Internet-Based Intervention Program lasting 300 mins |  |
| No preference | 49 (17.9.%) |
| Completion in 5 days (back-to-back), 60 mins per day | 101 (37%) |
| Completion in 5 weeks, 60 mins per week | 123 (45.1%) |
| Internet-Based Intervention 10 Sessions Program |  |
| No preference | 30 (11%) |
| One session per day | 102 (37.4%) |
| One session per week | 60 (22%) |
| Two sessions per week | 81 (29.7%) |
| Internet-Based Intervention - Time Limitation for Completion |  |
| No preference | 47 (17.2%) |
| No | 60 (22%) |
| Yes | 166 (60.8%) |
| Internet-Based Intervention Module Duration for One Sit |  |
| No preference | 38 (13.9%) |
| 15 mins | 80 (29.3%) |
| 30-45 mins | 128 (46.9%) |
| 45-60 mins | 27 (9.9%) |
| Internet-Based Intervention Module Presentation |  |
| No preference | 27 (9.9%) |
| A new module for everyday | 85 (31.1%) |
| A new module for every week | 148 (54.2%) |
| A new module for every month | 13 (4.8%) |
| Internet-Based Intervention Module Self-Assessment Scale/Questionnaire |  |
| No preference | 27 (9.9%) |
| No | 13 (4.8%) |
| Yes | 233 (85.3%) |
| Internet-Based Intervention Module Interaction Forms for Response Inputs |  |
| No preference | 39 (14.3%) |
| No | 17 (6.2%) |
| Yes | 217 (79.5%) |
| Internet-Based Intervention Continuation Reminder |  |
| No preference | 27 (9.9%) |
| Do not want a reminder | 7 (2.6%) |
| Everyday | 65 (23.8%) |
| Three times a week | 64 (23.4%) |
| Once a week | 110 (40.3%) |
| Internet-Based Intervention Program Character Identification |  |
| No preference | 45 (16.5%) |
| No | 23 (8.4%) |
| Yes | 205 (75.1%) |
| Internet Based Intervention Program-Homework |  |
| No preference | 46 (16.8%) |
| No | 53 (19.5%) |
| Yes | 174 (63.7%) |
| Internet-Based Intervention Program-Relaxation Exercises |  |
| No preference | 39 (14.3%) |
| No | 20 (7.3%) |
| Yes | 214 (78.4%) |
| ***Measurements form scales*** |  |
| Digital Literacy | 35.05 (8.74) |
| Anxiety | 10.13 (5.77) |
| Depressive Symptoms | 12.80 (6.63) |
| Attitudes toward guided internet interventions- perceived usefulness and helpfulness | 20.39 (5.45) |
| Attitudes toward guided internet interventions- perceived advantage relative to face-to-face therapy | 19.58 (7.47) |
| Attitudes toward guided internet interventions-total score | 39.97 (11.85) |
| ^1^Mean (SD); n (%) |  |
